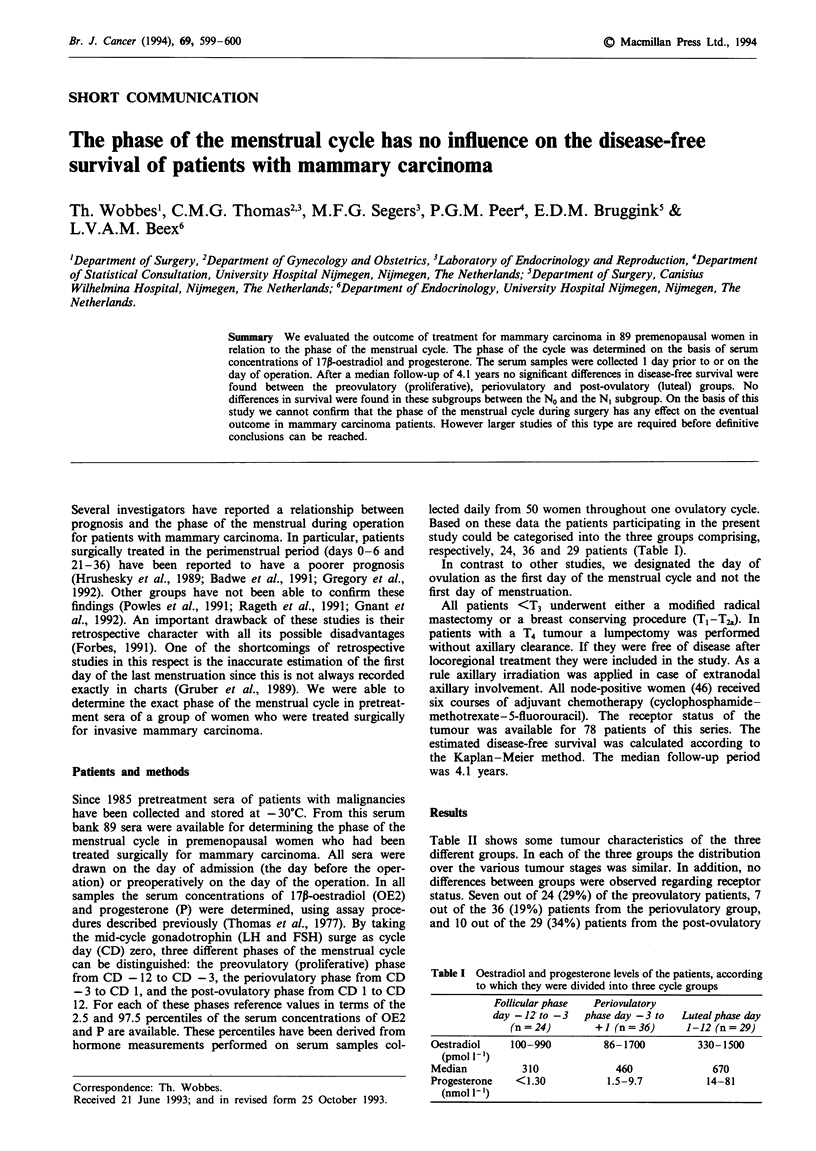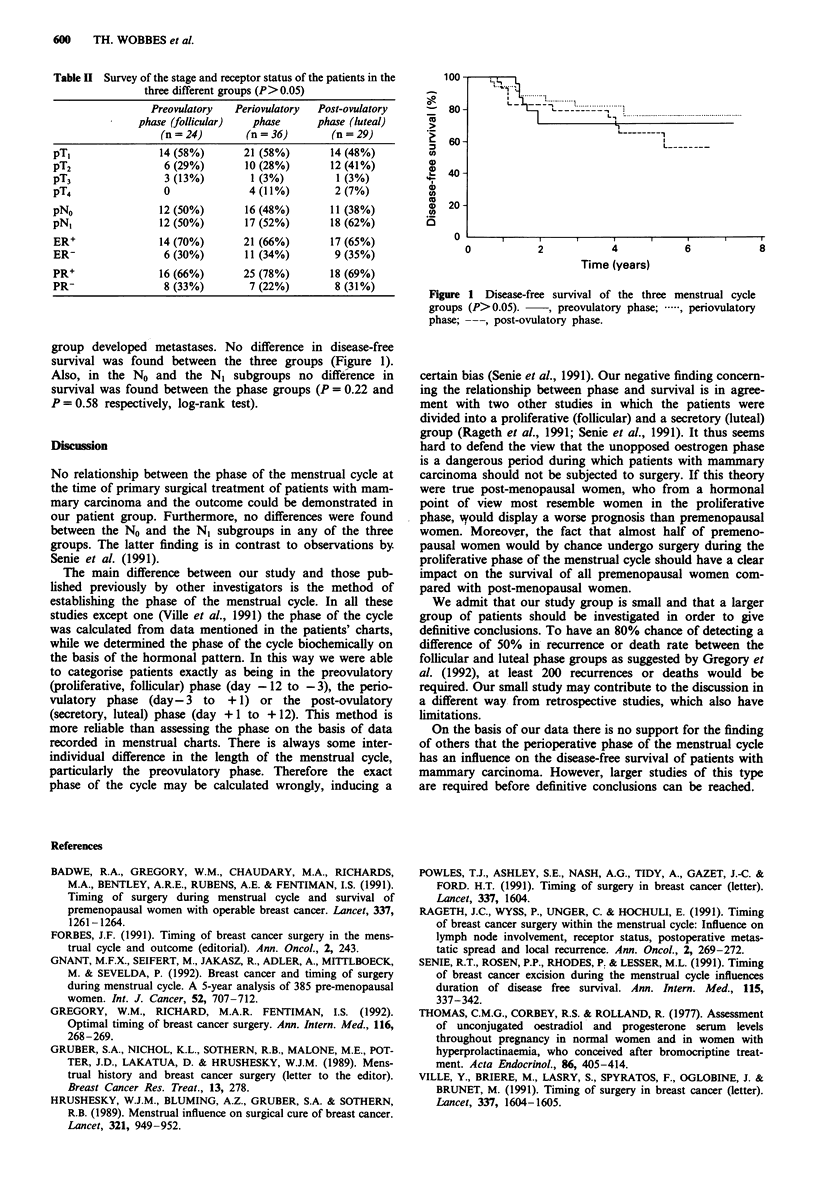# The phase of the menstrual cycle has no influence on the disease-free survival of patients with mammary carcinoma.

**DOI:** 10.1038/bjc.1994.110

**Published:** 1994-03

**Authors:** T. Wobbes, C. M. Thomas, M. F. Segers, P. G. Peer, E. D. Bruggink, L. V. Beex

**Affiliations:** Department of Surgery, University Hospital Nijmegen, The Netherlands.

## Abstract

We evaluated the outcome of treatment for mammary carcinoma in 89 premenopausal women in relation to the phase of the menstrual cycle. The phase of the cycle was determined on the basis of serum concentrations of 17 beta-oestradiol and progesterone. The serum samples were collected 1 day prior to or on the day of operation. After a median follow-up of 4.1 years no significant differences in disease-free survival were found between the preovulatory (proliferative), periovulatory and post-ovulatory (luteal) groups. No differences in survival were found in these subgroups between the N0 and N1 subgroup. On the basis of this study we cannot confirm that the phase of the menstrual cycle during surgery has any effect on the eventual outcome in mammary carcinoma patients. However larger studies of this type are required before definitive conclusions can be reached.


Br. J. Cancer (1994), 69, 599-600 © Macmillan Press Ltd., 1994

SHORT COMMUNICATION

### The phase of the menstrual cycle has no influence on the disease-free survival of patients with mammary carcinoma

Th. Wobbes[1], C.M.G. Thomas[2,3], M.F.G. Segers[3], P.G.M. Peer[4], E.D.M. Bruggink[5] & L.V.A.M. Beex[6]

[1]*Department of Surgery,* [2]*Department of Gynecology and Obstetrics,* [3]*Laboratory of Endocrinology and Reproduction,* [4]*Department of Statistical Consultation, University Hospital Nijmegen, Nijmegen, The Netherlands;* [5]*Department of Surgery, Canisius Wilhelmina Hospital, Nijmegen, The Netherlands;* [6]*Department of Endocrinology, University Hospital Nijmegen, Nijmegen, The Netherlands.*

**Summary** We evaluated the outcome of treatment for mammary carcinoma in 89 premenopausal women in relation to the phase of the menstrual cycle. The phase of the cycle was determined on the basis of serum concentrations of 17β-oestradiol and progesterone. The serum samples were collected 1 day prior to or on the day of operation. After a median follow-up of 4.1 years no significant differences in disease-free survival were found between the preovulatory (proliferative), periovulatory and post-ovulatory (luteal) groups. No differences in survival were found in these subgroups between the $N_0$ and the $N_1$ subgroup. On the basis of this study we cannot confirm that the phase of the menstrual cycle during surgery has any effect on the eventual outcome in mammary carcinoma patients. However larger studies of this type are required before definitive conclusions can be reached.

Several investigators have reported a relationship between prognosis and the phase of the menstrual during operation for patients with mammary carcinoma. In particular, patients surgically treated in the perimenstrual period (days 0-6 and 21-36) have been reported to have a poorer prognosis (Hrushesky *et al.*, 1989; Badwe *et al.*, 1991; Gregory *et al.*, 1992). Other groups have not been able to confirm these findings (Powles *et al.*, 1991; Rageth *et al.*, 1991; Gnant *et al.*, 1992). An important drawback of these studies is their retrospective character with all its possible disadvantages (Forbes, 1991). One of the shortcomings of retrospective studies in this respect is the inaccurate estimation of the first day of the last menstruation since this is not always recorded exactly in charts (Gruber *et al.*, 1989). We were able to determine the exact phase of the menstrual cycle in pretreatment sera of a group of women who were treated surgically for invasive mammary carcinoma.

### Patients and methods

Since 1985 pretreatment sera of patients with malignancies have been collected and stored at −30°C. From this serum bank 89 sera were available for determining the phase of the menstrual cycle in premenopausal women who had been treated surgically for mammary carcinoma. All sera were drawn on the day of admission (the day before the operation) or preoperatively on the day of the operation. In all samples the serum concentrations of 17β-oestradiol (OE2) and progesterone (P) were determined, using assay procedures described previously (Thomas *et al.*, 1977). By taking the mid-cycle gonadotrophin (LH and FSH) surge as cycle day (CD) zero, three different phases of the menstrual cycle can be distinguished: the preovulatory (proliferative) phase from CD −12 to CD −3, the periovulatory phase from CD −3 to CD 1, and the post-ovulatory phase from CD 1 to CD 12. For each of these phases reference values in terms of the 2.5 and 97.5 percentiles of the serum concentrations of OE2 and P are available. These percentiles have been derived from hormone measurements performed on serum samples collected daily from 50 women throughout one ovulatory cycle. Based on these data the patients participating in the present study could be categorised into the three groups comprising, respectively, 24, 36 and 29 patients (Table I).

In contrast to other studies, we designated the day of ovulation as the first day of the menstrual cycle and not the first day of menstruation.

All patients $<T_3$ underwent either a modified radical mastectomy or a breast conserving procedure ($T_1$-$T_{2a}$). In patients with a $T_4$ tumour a lumpectomy was performed without axillary clearance. If they were free of disease after locoregional treatment they were included in the study. As a rule axillary irradiation was applied in case of extranodal axillary involvement. All node-positive women (46) received six courses of adjuvant chemotherapy (cyclophosphamide-methotrexate-5-fluorouracil). The receptor status of the tumour was available for 78 patients of this series. The estimated disease-free survival was calculated according to the Kaplan-Meier method. The median follow-up period was 4.1 years.

### Results

Table II shows some tumour characteristics of the three different groups. In each of the three groups the distribution over the various tumour stages was similar. In addition, no differences between groups were observed regarding receptor status. Seven out of 24 (29%) of the preovulatory patients, 7 out of the 36 (19%) patients from the periovulatory group, and 10 out of the 29 (34%) patients from the post-ovulatory

**Table I** Oestradiol and progesterone levels of the patients, according to which they were divided into three cycle groups

| | *Follicular phase day −12 to −3* (n = *24)* | *Periovulatory phase day −3 to +1* (n = *36)* | *Luteal phase day 1-12* (n = *29)* |
|---|---|---|---|
| Oestradiol (pmol l$^{-1}$) | 100-990 | 86-1700 | 330-1500 |
| Median | 310 | 460 | 670 |
| Progesterone (nmol l$^{-1}$) | <1.30 | 1.5-9.7 | 14-81 |

Correspondence: Th. Wobbes.
Received 21 June 1993; and in revised form 25 October 1993.

**Table II** Survey of the stage and receptor status of the patients in the three different groups ($P > 0.05$)

| | *Preovulatory phase (follicular)* (n = *24*) | *Periovulatory phase* (n = *36*) | *Post-ovulatory phase (luteal)* (n = *29*) |
|---|---|---|---|
| $pT_1$ | 14 (58%) | 21 (58%) | 14 (48%) |
| $pT_2$ | 6 (29%) | 10 (28%) | 12 (41%) |
| $pT_3$ | 3 (13%) | 1 (3%) | 1 (3%) |
| $pT_4$ | 0 | 4 (11%) | 2 (7%) |
| $pN_0$ | 12 (50%) | 16 (48%) | 11 (38%) |
| $pN_1$ | 12 (50%) | 17 (52%) | 18 (62%) |
| $ER^+$ | 14 (70%) | 21 (66%) | 17 (65%) |
| $ER^-$ | 6 (30%) | 11 (34%) | 9 (35%) |
| $PR^+$ | 16 (66%) | 25 (78%) | 18 (69%) |
| $PR^-$ | 8 (33%) | 7 (22%) | 8 (31%) |

**Figure 1** Disease-free survival of the three menstrual cycle groups ($P > 0.05$). ——, preovulatory phase; ·····, periovulatory phase; - - -, post-ovulatory phase.

group developed metastases. No difference in disease-free survival was found between the three groups (Figure 1). Also, in the $N_0$ and the $N_1$ subgroups no difference in survival was found between the phase groups ($P = 0.22$ and $P = 0.58$ respectively, log-rank test).

### Discussion

No relationship between the phase of the menstrual cycle at the time of primary surgical treatment of patients with mammary carcinoma and the outcome could be demonstrated in our patient group. Furthermore, no differences were found between the $N_0$ and the $N_1$ subgroups in any of the three groups. The latter finding is in contrast to observations by Senie *et al.* (1991).

The main difference between our study and those published previously by other investigators is the method of establishing the phase of the menstrual cycle. In all these studies except one (Ville *et al.*, 1991) the phase of the cycle was calculated from data mentioned in the patients' charts, while we determined the phase of the cycle biochemically on the basis of the hormonal pattern. In this way we were able to categorise patients exactly as being in the preovulatory (proliferative, follicular) phase (day $-12$ to $-3$), the periovulatory phase (day $-3$ to $+1$) or the post-ovulatory (secretory, luteal) phase (day $+1$ to $+12$). This method is more reliable than assessing the phase on the basis of data recorded in menstrual charts. There is always some interindividual difference in the length of the menstrual cycle, particularly the preovulatory phase. Therefore the exact phase of the cycle may be calculated wrongly, inducing a certain bias (Senie *et al.*, 1991). Our negative finding concerning the relationship between phase and survival is in agreement with two other studies in which the patients were divided into a proliferative (follicular) and a secretory (luteal) group (Rageth *et al.*, 1991; Senie *et al.*, 1991). It thus seems hard to defend the view that the unopposed oestrogen phase is a dangerous period during which patients with mammary carcinoma should not be subjected to surgery. If this theory were true post-menopausal women, who from a hormonal point of view most resemble women in the proliferative phase, would display a worse prognosis than premenopausal women. Moreover, the fact that almost half of premenopausal women would by chance undergo surgery during the proliferative phase of the menstrual cycle should have a clear impact on the survival of all premenopausal women compared with post-menopausal women.

We admit that our study group is small and that a larger group of patients should be investigated in order to give definitive conclusions. To have an 80% chance of detecting a difference of 50% in recurrence or death rate between the follicular and luteal phase groups as suggested by Gregory *et al.* (1992), at least 200 recurrences or deaths would be required. Our small study may contribute to the discussion in a different way from retrospective studies, which also have limitations.

On the basis of our data there is no support for the finding of others that the perioperative phase of the menstrual cycle has an influence on the disease-free survival of patients with mammary carcinoma. However, larger studies of this type are required before definitive conclusions can be reached.

### References

BADWE, R.A., GREGORY, W.M., CHAUDARY, M.A., RICHARDS, M.A., BENTLEY, A.R.E., RUBENS, A.E. & FENTIMAN, I.S. (1991). Timing of surgery during menstrual cycle and survival of premenopausal women with operable breast cancer. *Lancet*, **337**, 1261–1264.

FORBES, J.F. (1991). Timing of breast cancer surgery in the menstrual cycle and outcome (editorial). *Ann. Oncol.*, **2**, 243.

GNANT, M.F.X., SEIFERT, M., JAKASZ, R., ADLER, A., MITTLBOECK, M. & SEVELDA, P. (1992). Breast cancer and timing of surgery during menstrual cycle. A 5-year analysis of 385 pre-menopausal women. *Int. J. Cancer*, **52**, 707–712.

GREGORY, W.M., RICHARD, M.A.R. FENTIMAN, I.S. (1992). Optimal timing of breast cancer surgery. *Ann. Intern. Med.*, **116**, 268–269.

GRUBER, S.A., NICHOL, K.L., SOTHERN, R.B., MALONE, M.E., POTTER, J.D., LAKATUA, D. & HRUSHESKY, W.J.M. (1989). Menstrual history and breast cancer surgery (letter to the editor). *Breast Cancer Res. Treat.*, **13**, 278.

HRUSHESKY, W.J.M., BLUMING, A.Z., GRUBER, S.A. & SOTHERN, R.B. (1989). Menstrual influence on surgical cure of breast cancer. *Lancet*, **321**, 949–952.

POWLES, T.J., ASHLEY, S.E., NASH, A.G., TIDY, A., GAZET, J.-C. & FORD. H.T. (1991). Timing of surgery in breast cancer (letter). *Lancet*, **337**, 1604.

RAGETH, J.C., WYSS, P., UNGER, C. & HOCHULI, E. (1991). Timing of breast cancer surgery within the menstrual cycle: Influence on lymph node involvement, receptor status, postoperative metastatic spread and local recurrence. *Ann. Oncol.*, **2**, 269–272.

SENIE, R.T., ROSEN, P.P., RHODES, P. & LESSER, M.L. (1991). Timing of breast cancer excision during the menstrual cycle influences duration of disease free survival. *Ann. Intern. Med.*, **115**, 337–342.

THOMAS, C.M.G., CORBEY, R.S. & ROLLAND, R. (1977). Assessment of unconjugated oestradiol and progesterone serum levels throughout pregnancy in normal women and in women with hyperprolactinaemia, who conceived after bromocriptine treatment. *Acta Endocrinol.*, **86**, 405–414.

VILLE, Y., BRIERE, M., LASRY, S., SPYRATOS, F., OGLOBINE, J. & BRUNET, M. (1991). Timing of surgery in breast cancer (letter). *Lancet*, **337**, 1604–1605.